# The Diagnostic Value of Cellular Phenotyping and Pathological Casts Using Urine Flow Cytometry in Children with Lupus Nephritis

**DOI:** 10.3390/diseases14020053

**Published:** 2026-02-01

**Authors:** Ferdy Royland Marpaung, Risky Vitria Prasetyo, Anggia Augustasia Lumban Toruan, Djoko Santoso, Aryati Aryati

**Affiliations:** 1 Doctoral Program of Medical Science, Faculty of Medicine, Universitas Airlangga, Surabaya 60131, Indonesia; ferdy.royland.marpaung-2022@fk.unair.ac.id; 2 Dr. Soetomo General Academic Hospital, Surabaya 60286, Indonesia; risky-v-p@fk.unair.ac.id (R.V.P.); djoko-santoso@fk.unair.ac.id (D.S.); 3 Department of Child Health, Faculty of Medicine, Universitas Airlangga, Surabaya 60131, Indonesia; 4 Departement of Urology, Gotong Royong General Hospital, Surabaya 60119, Indonesia; anggia05@gmail.com; 5 Department of Internal Medicine, Faculty of Medicine, Universitas Airlangga, Surabaya 60131, Indonesia; 6 Department of Clinical Pathology, Faculty of Medicine, Universitas Airlangga, Surabaya 60131, Indonesia

**Keywords:** lupus nephritis, erythrocyte dysmorphic, renal tubular epithelial cell, pathological cast, flow cytometry, non-communicable disease

## Abstract

Introduction: Dysmorphic RBC (DysRBC) as a marker of glomerular abnormalities is expected to have added value in screening for glomerular abnormalities along with other examinations, including renal tubular epithelial cells (RTECs) and pathological casts (PathCasts) that indicate tubular abnormalities in lupus nephritis (LN). Therefore, this study intended to assess the diagnostic performance of urinary cell and cast characteristics, including DysRBC, RTECs, and PathCast, as measured by the urine flow cytometry in lupus nephritic children. Methods: Urine samples from 317 patients (50.47% female and 49.53% male) were collected. The diagnostic value was evaluated using receiver operating characteristic (ROC) analysis. Results: The ROC analysis demonstrated that all parameters exhibited acceptable discriminatory performance, including %DysRBC (AUC = 0.954, *p* < 0.001), RTEC (AUC = 0.580, *p* = 0.001), and PathCast (AUC = 0.664, *p* = 0.001). Conclusions: DysRBC, RTECs, and PathCast may have added value in the diagnosis of LN in children, notably with excellent diagnostic value in distinguishing LN in %DysRBC. This promising result warrants evaluation with a large-scale site study.

## 1. Introduction

Lupus nephritis (LN) represents a serious renal manifestation of systemic lupus erythematosus (SLE) [1] and remains a major contributor to morbidity and mortality, particularly in pediatric patients [2]. Although childhood-onset LN is relatively uncommon, it is often characterized by a more aggressive clinical course and more severe renal involvement compared with adult-onset disease. Early stages of LN may present with subtle or nonspecific clinical findings; however, progressive immune-mediated renal injury can ultimately lead to irreversible kidney damage and end-stage renal disease. Consequently, the identification of sensitive, non-invasive markers for early renal involvement is essential to improve outcomes in children with SLE [3]. Current approaches for evaluating renal involvement in SLE include serial measurement of serum creatinine, assessment of urinary protein excretion using the albumin-to-creatinine ratio (ACR), and microscopic examination of urine sediment. While these methods are widely used, they may lack sufficient sensitivity for early detection or monitoring of disease activity. Urinary cellular phenotypes, such as dysmorphic red blood cells (DysRBCs), renal tubular epithelial cells (RTECs), and pathological casts (PathCasts), reflect distinct sites of renal injury and may provide additional diagnostic information. DysRBCs are considered markers of glomerular damage, whereas RTECs and PathCasts indicate tubular and combined glomerulotubular injury, respectively [4].

Advances in urine sediment analysis using automated flow cytometry have enabled more precise and reproducible identification of urinary cellular components, including dysRBC, RTEC, and PathCasts. While these parameters have been individually associated with glomerular or tubular injury, their combined diagnostic performance using automated urine flow cytometry has not been well established in pediatric lupus nephritis [5]. Most existing studies focus on adult populations, manual microscopy, or isolated urinary markers, limiting their applicability to children. Therefore, this study aimed to evaluate the diagnostic value and optimal cut-off points of %DysRBC, RTEC, and PathCast measured by urine flow cytometry in children with lupus nephritis, providing a non-invasive and clinically applicable approach for screening and an adjunctive tool.

## 2. Materials and Methods

### 2.1. Subjects

This was an observational study conducted from 2024 to 2025 at Dr. Soetomo Hospital, Surabaya, Indonesia, involving 317 children. Of these, 221 were diagnosed with LN and had undergone kidney histopathology, and 96 patients were diagnosed with non-LN. Of the 96 non-LN children, 90 were diagnosed with lower UTI (cystitis and urethritis), and 6 with ureteral stones. The lupus nephritis classification system was established based on the latest guidelines from the WHO and ISN/RPS (International Society of Nephrology/Renal Pathology Society), encompassing six classes [6]. Routine tests, such as blood creatinine and urine albumin–creatinine ratio, were performed using a Mindray BS 120 chemistry instrument (Mindray Corporation, Shenzhen, China). Creatinine in blood and urine was tested using the enzymatic method; microalbuminuria was tested using quantitative immunoturbidimetry (microalbumin–turbilatex). The albumin–creatinine ratio (ACR) was classified into 3 groups (ACR < 30 mg/g, ACR = 30–300 mg/g, and ACR > 300 mg/g).

### 2.2. Urine Flow Cytometry

Urine sediment analysis was performed using first-morning midstream urine samples collected immediately prior to the kidney biopsy. A volume of 25–50 mL of fresh urine was analyzed within 2 h of collection without centrifugation to minimize cellular degradation. Samples were processed using the Sysmex UF-5000 urine flow cytometer (Sysmex Corporation, Kobe, Japan) according to the manufacturer’s instructions. The UF-5000 employs hydrodynamic focusing and a blue semiconductor laser to analyze urinary particles based on forward scatter, side scatter, fluorescence intensity, and depolarized side scatter. Dysmorphic red blood cells (%DysRBC) were identified based on characteristic alterations in light scatter and fluorescence signals reflecting changes in cell size and membrane morphology, and are reported as a percentage of total red blood cells. RTEC and PathCast were identified and quantified using predefined, manufacturer-validated gating algorithms and reported as elements per microliter (/µL). Quality control and calibration were performed daily according to laboratory standards.

Automated urine flow cytometry was selected instead of phase contrast microscopy because it provides a standardized, objective, and reproducible quantitative assessment of urinary cellular components with minimal operator dependence, making it suitable for routine evaluation and large pediatric cohorts.

Due to the use of proprietary, manufacturer-validated algorithms in the Sysmex UF-5000 system, raw dot plots or histograms cannot be exported for external visualization. This limitation reflects the technical constraints of the analyzer software rather than a methodological omission.

### 2.3. Ethical Clearance

The Institutional Review Board of Dr. Soetomo Academic Hospital, Surabaya, Jawa Timur, Indonesia, approved this study (03988/KEPK/III/2024). The research was conducted in compliance with the Declaration of Helsinki (2013). The patients, parents, or relatives gave their written informed consent to be involved in this study.

### 2.4. Statistical Analysis

The Kolmogorov–Smirnov test was performed for the normality test. The diagnostic value of %DysRBC, RTEC, and PathCast LN was calculated with ROC curve analysis. In this analysis, the sensitivity, specificity, cut-off, and area under the curve (AUC) were estimated to distinguish between lupus nephritis and non-lupus nephritis. An AUC of 0.5 signifies a lack of discrimination, whereas values of 0.5–0.6 were deemed poor. An AUC of 0.6–0.7 was considered fair, 0.7–0.8 was acceptable, 0.8–0.9 was classified as excellent, and finally, values > 0.9 were deemed exceptional [7]. Categorical variables were compared using the chi-square test, while continuous variables were compared using the Kruskal–Wallis test. The Kruskal–Wallis test was used for non-normally distributed variables, while ANOVA was applied to assess the contribution of %DysRBC, RTEC, and PathCast to LN classification. The significance level of *p* < 0.05 was respected as statistically significant. All statistical analyses were performed using MedCalc^®^ software for Windows version 20.218 (Ostend, Belgium).

## 3. Results

In a cohort of 317 patients, 160 (50.47%) were female, and 157 (49.53%) were male, with a median age of 13 years. Table 1 shows a comprehensive summary of patient characteristics. The predominant LN classification observed was Class IV, while Class V was the least frequently encountered (Table 1).

Blood creatinine levels, urine albumin–creatinine ratio, %DysRBC, RTECs, and PathCast exhibited significant differences between LN and non-LN patients, with all parameters demonstrating higher values in LN patients compared to non-LN (Table 2).

Notable differences were identified in %DysRBC, RTEC, and PathCast according to the ACR classification, with ACR > 30 mg/g correlating with elevated %DysRBC, RTEC, and PathCast in comparison to ACR < 30 mg/g (Table 2).

Two-thirds of participants exhibited a normal ACR (<30 mg/g), whereas cases with abnormal ACR (>30 mg/g) were more common among LH patients (*p* < 0.001) (Table 3).

**Figure 1:**
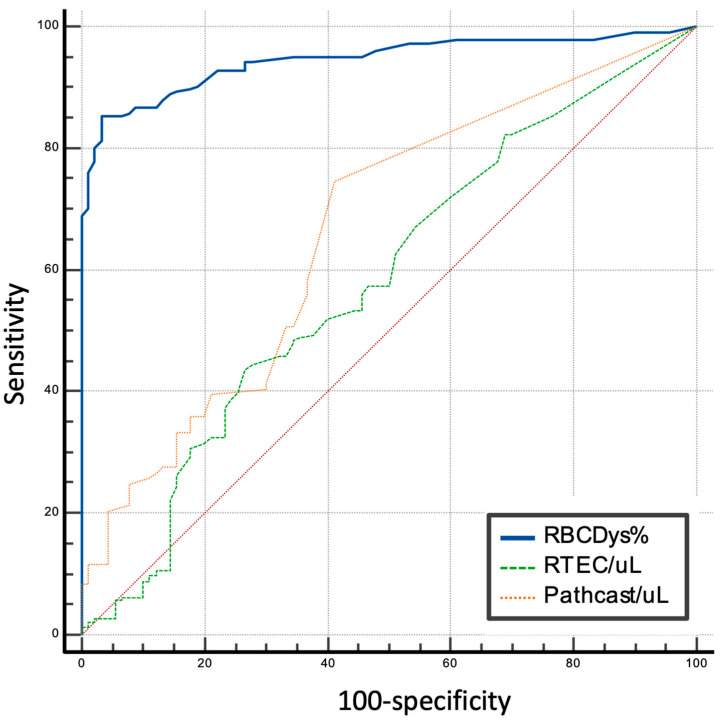
Receiver operating characteristic (ROC) curves illustrating the diagnostic performance of urinary biomarkers measured by urine flow cytometry, including the %DysRBC, RTEC, and PathCast, in distinguishing LN. The area under the curve (AUC) is shown for each parameter, demonstrating their ability to discriminate LN at 100% specificity.

The %DysRBC parameter demonstrated significant diagnostic value, exhibiting high sensitivity and specificity in distinguishing LN from non-LN, with an AUC of 94.5% with a cut-off value 35%. In contrast, RTEC was poor, and PathCast displayed fair AUC values (0.580 and 0.664, respectively). Table 4 and Figure 1 provide the optimal cut-off values and AUC for each parameter.

## 4. Discussion

SLE primarily impacts female children, with 15% to 20% of cases diagnosed prior to age 18 [8]. This study identified similar demographics in which 50.5% of the population was female. A previous study showed that childhood-onset systemic lupus erythematosus (SLE) is generally more aggressive, with increased rates of renal involvement leading to LN and other notable consequences, such as hematological issues and neuropsychiatric effects [9]. LN is a notable complication, with age influencing both clinical presentation and disease severity. Recent studies suggest that pediatric patients could achieve favorable long-term renal outcomes if treated immediately [10]. Therefore, routine screening and monitoring are essential for the early detection of LN [11].

Advancements in urine sediment testing, capable of identifying cells and various components in urine, are advancing swiftly [5]. At present, two methodologies exist: flow cytometry and digital imaging. Flow cytometry utilizes a hydrodynamic focusing system to identify and separate elements in urine sediment [5]. In this method, the blue semiconductor laser beam is emitted, resulting in side and forward scatter, depolarized side scatter, and side fluorescence upon interaction with cells or elements in the urine. Stratification is subsequently conducted based on the size, complexity, and nucleic acid content of the elements. Other methods, including digital imaging, have incorporated artificial intelligence through validated algorithms [12]. In this study, we evaluated the research parameter flow cytometry urinalysis of DysRBC and routine reported parameters that were recently available in our routine laboratory, including RTEC and PathCast.

This study demonstrates that the proportion of dysmorphic red blood cells measured by urine flow cytometry shows excellent diagnostic performance for distinguishing pediatric lupus nephritis from non-lupus nephritis cases. Among the evaluated urinary parameters, %DysRBC exhibited the highest sensitivity, specificity, and area under the ROC curve, highlighting its potential value as a non-invasive screening and adjunctive monitoring tool in children with SLE.

This study’s findings indicate that the optimal cut-off value of 35% DysRBC demonstrates high sensitivity and specificity and may serve as a benchmark for identifying glomerular damage in children with LN. This cut-off may be significant for clinicians to promote more intensive treatment approaches and is anticipated to be applicable in therapy monitoring.

Variability in cut-off values across studies may be attributed to differences in patient age, underlying disease severity, analytical methods, and histopathological distribution. The range varied from 20% to 80% [13,14,15,16]. For instance, the study by Setiawan D et al. identified 68% DysRBC as the optimal cut-off value for differentiating glomerular from non-glomerular conditions in adult and children hematuria patients, finding sensitivity and specificity of 91% and 85%, respectively, and an AUC of 0.890 [13]. Several studies also indicate that %DysRBC varied based on the extent of glomerular damage [17,18,19,20]. In the present cohort, class IV lupus nephritis was predominant. This diffuse form of LN is characterized by extensive immune complex deposition and glomerular inflammation, which may result in a higher proportion of dysmorphic erythrocytes due to severe disruption of the glomerular filtration barrier. These findings suggest that %DysRBC thresholds may vary according to disease activity and histological class, warranting further investigation in larger and more evenly distributed cohorts. Currently, the research on the diagnostic value of %DysRBC in lupus nephritis in children is insufficient. Nevertheless, the diagnostic significance of dysmorphic RBCs is established as highly effective in identifying glomerular damage [21]. Red blood cells (RBCs) are shed while traversing the basement membrane, and the influence of podocytes leads to the formation of atypical RBCs, including D1, D2, and D3 [22]. These shapes possess distinct dimensions relative to standard red blood cells. The proportion of dysmorphic cells is referred to as %DysRBC. Our previous study showed an excellent concordance of flow cytometry to phase contrast in the detection of glomerular hematuria [23].

Another finding in this study indicated a poor AUC of RTEC. RTECs play a crucial role in tubular damage. Systemic inflammation targets RTEC, resulting in acute kidney injury [24,25]. Tubular cells are involved in the reaction to mediators of inflammation in ischemic and septic kidney injury [26]. Their presence in urine suggests the occurrence of tubular damage [27,28]. Furthermore, the loss of tubular cells through epithelial-to-mesenchymal transition or apoptosis may create conditions conducive to the development of chronic kidney disease (CKD). RTECs are typically difficult to detect in sediment. These variables may explain the poor diagnostic value of RTEC, as finding intact RTEC may not adequately represent the condition.

Finally, this study revealed the fair performance of PathCasts in distinguishing the LN. PathCast results from diminished urine flow caused by injury to the glomerulus and tubules. Pathological cylinders comprise the inclusions of particles, including red blood cells in RBC cylinders, white blood cells in WBC cylinders, RTECs in epithelial cylinders, granular components in granular cylinders, fat droplets in fatty cylinders, and others [29]. However, these pathological cylinders are characterized by fragility and susceptibility to damage [30]. This phenomenon may explain the inferior diagnostic value compared to the %DysRBC parameter. Zhang et al. suggested that the urinary microprotein test ought to be incorporated into the re-evaluation procedures for routine assessments of patients exhibiting negative protein results alongside positive casts in microscopic evaluations [31]. In addition, RTECs are fragile and may rapidly degrade in urine, while PathCasts are highly dependent on urine flow, sample timing, and pre-analytical handling. These biological and technical factors likely reduce their diagnostic reliability compared with the more stable DysRBC parameter.

Importantly, this study provides pediatric-specific diagnostic cut-off values for %DysRBC derived from automated urine flow cytometry, addressing a critical gap in current non-invasive lupus nephritis screening strategies.

## 5. Limitations

Despite the importance of the results of this study, several limitations must be considered. First, non-LN patients were not identified based on biopsy, which may have introduced bias. However, due to clinical practice and ethical issues, a biopsy was not feasible in these circumstances. Kidney biopsy in non-glomerular cases may lead to glomerular damage. Secondly, this study was a single-center evaluation from tertiary hospitals; therefore, further research on a larger scale is needed to generalize the results and ensure external validation. Finally, a detailed analysis of patient comorbidities, including prior COVID-19 infection, was not performed and may represent a potential confounding factor in the interpretation of urinary cellular findings.

## 6. Conclusions

The use of the proportion of dysmorphic RBCs demonstrated high diagnostic value in determining LN, compared with RTEC and PathCast parameters. Further research on a larger scale and in multiple sites is expected in the future.

## Figures and Tables

**Table 1:** Participants’ demographics.

Characteristic	Result
Sex (n, %)	
Male	160 (50.5%)
Female	157 (49.5%)
Age (years)	
Median (IQR)	13 (7)
BW (kg)	
Median (IQR)	36.6 (22)
Height (cm)	
Median (IQR)	145 (32)
LN classification system (n, %)	
Class I	8 (3.6%)
Class II	16 (7.2%)
Class III	56 (25.3%)
Class IV	124 (56.1%)
Class V	7 (3.2%)
Class VI	10 (4.6%)
ACR (n, %)	
<30	211 (66.6)
30–300	42 (13.2)
>300	64 (20.2)

Note: LN: lupus nephritis, IQR: interquartile range, ACR: albumin–creatinine ratio.

**Table 2:** Characteristics of comparison parameters in LN and non-LN.

Characteristic	Result	*p* Value
Blood Creatinine (mg/dL)		
Median (IQR)	0.63 (0.4)	
LN Median (IQR)	0.67 (0.39)	0.021 *
Non-LN Median (IQR)	0.57 (0.32)	
Urine ACR (mg/g) Median (IQR)		
Total	9.9 (173.6)	
LN	14.8 (304.4)	<0.001 *
Non-LN	4.3 (9.5)	
%DysRBC		
ACR < 30	39	
ACR 30–300	56	0.022 *
ACR > 300	59	
RTEC/uL		
ACR < 30	0.6 (1.9)	
ACR 30–300	1.5 (4.0)	0.006 *
ACR > 300	1.7 (3.9)	
PathCast (/µL)		
ACR < 30	0.01 (0.4)	
ACR 30–300	0.27 (1.4)	0.002 *
ACR > 300	0.41 (1.2)	
%DysRBC (median, IQR)		
Total	48 (52.5)	
LN	60 (32)	<0.001 *
Non-LN	8 (14)	
RTEC (/µL)		
Total Median (IQR)	1.4 (3.43)	
LN Median (IQR)	1.5 (3.5)	0.0258 *
Non-LN Median (IQR)	0.95 (2.2)	
PathCast (/µL)		
Total Median (IQR)	0.27 (1.34)	
LN Median (IQR)	0.41 (1.84)	<0.001 *
Non-LN	0.01 (0.5)	

* Kruskal–Wallis, LN: lupus nephritis, IQR: interquartile range, ACR: albumin–creatinine ratio.

**Table 3:** Contingency urine albumin–creatinine ratio classification and diagnosis.

	ACR Classification			Total (n, %)
Diagnosis	<30	30–300	>300	
Non-LN	77	10	3	90 (28.4)
LN	134	32	61	227 (71.6%)
Total (n, %)	211 (66.6%)	42 (13.2%)	64 (20.2%)	
*p* value < 0.001 *				

* Chi-square, ACR: albumin–creatinine ratio, LN: lupus nephritis.

**Table 4:** The diagnostic value and cut-off of %DysRBC, RTEC, and PathCast in distinguishing LN.

Parameter	AUC	Sens	Spes	Cut off	*p* Value
%DysRBC	0.945	84.6	96.7	>35	<0.0001
RTEC (/uL)	0.580	43.17	73.33	>2.2	<0.001
PathCast (/uL)	0.664	74.67	58.89	>0.13	<0.001

## Data Availability

The datasets presented in this article are not readily available because the data are part of an ongoing study. Requests to access the datasets should be directed to aryati@gmail.com.
